# Systems Thinking: Identifying Leverage Points for Achieving a Continuum of Competency-Based Health Professions Education

**DOI:** 10.5334/pme.2573

**Published:** 2026-09-29

**Authors:** Karen E. Hauer, Arvin K. Damodaran, Jason R. Frank, Soeren Huwendiek, Adrian P. Marty, Denyse Richardson, Daniel J. Schumacher, Pedro Tanaka, Teri Lee Turner, Denise H. Rhoney, For The International Competency-Based Health Professions Education Collaborators

**Affiliations:** 1 Department of Medicine, University of California, San Francisco (UCSF), San Francisco, California, USA; 2 School of Clinical Medicine, Faculty of Medicine and Health, UNSW Sydney, Australia; 3 Centre for Innovation in Medical Education, University of Ottawa, Ottawa, Canada; 4 The Ottawa Hospital, Ottawa, Canada; 5 Department for Assessment and Evaluation, Institute for Medical Education in Bern, University of Bern, Switzerland; 6 Department of Anesthesiology, Intensive Care Medicine and Pain Therapy, University Hospital Balgrist, Zurich, Switzerland; 7 Division of PMR (Physiatry), Department of Medicine, University of Alberta, Edmonton, Alberta, Canada; 8 Department of Pediatrics at Cincinnati Children’s Hospital Medical Center and University of Cincinnati College of Medicine in Cincinnati, Ohio, USA; 9 Stanford University School of Medicine, Stanford, California, USA; 10 Department of Pediatrics at Baylor College of Medicine, Houston, Texas, USA; 11 UNC Eshelman School of Pharmacy, CB 7574, Chapel Hill, NC 27599-7574, USA

## Abstract

Competency-based education is widely endorsed in health professions education (CBHPE) as an outcomes-focused approach to ensuring that all graduates demonstrate the competencies needed to improve patient and population health. Despite adoption of outcomes frameworks, accreditation requirements, and CBHPE tactics, interventions remain siloed within programs and training levels rather than spanning the education to practice continuum. Consequently, learners and teachers experience fragmented educational journeys that can fall short of addressing each learner’s needs for continuous learning and growth. To advance efforts toward a CBHPE continuum, this systems-informed conceptual analysis uses a systems thinking perspective to analyze leverage points, places within complex systems where small, strategic interventions can cause large-scale, lasting changes. Drawing on Meadows’ framework for understanding leverage points in complex systems, we examined barriers and opportunities to achieve a continuum and present five theoretically informed, literature-based, and expert-derived recommendations for transformation.

Our analysis revealed that strategic intervention points, including paradigm shifts from ‘time-served’ to ‘competence-demonstrated’ readiness, structural changes through cross-continuum governance alignment, and process modifications via shared competency language and data systems, enable meaningful transformation beyond surface-level adjustments. Key recommendations include: (1) restructuring system architecture to enable seamless learner progression through competency committees and joint governance bodies; (2) shifting mental models to normalize time-variable progression and reconceptualize assessment as growth-supporting; (3) implementing high-impact approaches to assessment with longitudinal learner analytics; (4) changing rules and incentives to reward competency achievement; and (5) establishing continuous data-driven feedback systems. Understanding these leverage points informs targeted approaches, enabling a cohesive, coordinated, learner-centered CBHPE continuum.

## Introduction

A coordinated continuum of competency-based health professions education (CBHPE) is needed because healthcare professionals learn and provide care concurrently throughout their careers, making education and practice inseparable for high-quality patient care [1,2]. CBHPE emphasizes outcomes, developmental progression, and individualized learning [3,4]. However, most implementations remain confined to discrete stages of training rather than spanning the full educational continuum into practice [5,6]. Consequently, the potential of CBHPE to support continuous professional development and system-level improvement has not been fully realized [7]. CBHPE emphasizes defined outcomes and individualized, time-variable progression. However, time-variable progression is not possible in many settings due to administrative requirements and rules, fixed time structures, and resistance to changing current approaches [3,8]. Alignment across transitions is necessary to support learners’ progression toward outcomes and readiness for practice.

Attention to the continuum of CBHPE addresses these limitations by reducing fragmentation, which disrupts progression and may compromise the quality of care. The continuum entails lifelong learning across “the career from the prelicensure learner to fully independent practice” [9]. For learners, CBHPE has the potential to eliminate artificial boundaries between training stages, support clear developmental pathways, and minimize redundancy across disconnected educational experiences [5]. A CBHPE continuum recognizes that competence must be continuously developed and sustained across training phases and into a professional career, aligning educational structures with the evolving demands of healthcare and contributing to improved patient outcomes.

This manuscript focuses on the CBHPE continuum as a system-level construct. Although CBHPE is widely described as an educational approach, it is less often conceptualized or implemented as an integrated system spanning the full trajectory of professional development. We define the CBHPE continuum as a coordinated system in which learners progress across all stages based on demonstrated competence. This progression is supported by aligned assessment, data continuity, and interconnected educational structures. Whereas CBHPE describes the approach [10], the CBHPE continuum represents its system-level implementation and coordination.

To address the gap between how CBHPE is currently implemented and how it could be implemented as a system across the continuum of care, we apply a systems-thinking lens. We draw on Meadows’ leverage points framework [11], described below, to examine the structural and cultural factors that sustain fragmentation and to identify high-impact opportunities for change. The purpose of this manuscript is to (1) define the key features of a CBHPE continuum, (2) identify barriers that impede its realization, and (3) propose leverage points for advancing a more integrated and effective system. By doing so, we offer a framework that links conceptual understanding with actionable strategies for advancing a CBHPE continuum.

## Historical Philosophies and Paradigms that Contribute to the Current State

Several fundamental historical philosophies and paradigms underlie barriers to a continuum in HPE. The industrial factory model of education originating in the early 19th century emphasized standardization, efficiency, and time-based cohort progression, concepts that persist in HPE’s structures [12]. The Flexner Report of 1910, which drew on German approaches and addressed medical education in the United States and Canada, transformed medical education worldwide, with its recommendations for structure- and process-based education design [13,14,15]. Standardized curricula, fixed durations, and institution-based training became embedded across the medical and, consequently, other health professions. The concept of educational phases emerged with separate rules, governance, standards, gatekeeping mechanisms, and regulatory bodies. Views of HPE as workforce production with system goals to meet societal needs and trainee availability to meet clinical service needs also drove time-based and efficiency-focused approaches over individual competency development [16].

Historical understanding of assessment paradigms also explains barriers to a CBHPE continuum. The psychometric paradigm positions assessment as a measurement problem, with the aim of telling people apart rather than supporting learning or individualized progression [17]. By contrast, an assessment *for* learning or developmental assessment serves a learning and development function and supports individual progression through one’s career. The desire for ‘objective’ assessment methods, borrowed from scientific measurement traditions, has led to over-reliance on quantifiable metrics and standardized approaches [18]. The checklist approach that emerged from aviation safety models and industrial quality control has reduced complex clinical performance and assessment of competence to discrete, measurable items [19]. Taken together, these historical factors set the stage for a fragmented rather than continuous experience of CBHPE.

### Analytic approach

This author group, all members of the International Competency-Based HPE collaborative (ICBHPE) and experts in CBHPE implementation and scholarship, previously recognized longstanding shortcomings in the CBHPE continuum. The analytic process for this manuscript entailed first conducting a literature review on CBHPE, transitions across CBHPE phases, and the history of education paradigms that inform CBHPE. A consistent theme across the HPE literature is that fundamental system-level issues must be addressed to improve CBHPE. Ten Cate (2019) argues for a shift from siloed, time-bound training toward a continuum of competency development across the career, requiring new mental models of lifelong learning and competency management [18]. Similarly, Frank et al. (2017) emphasize that meaningful progress toward implementing CBHPE with fidelity and achieving acceptance by educators, learners, and leaders depends on changes in professional and organizational culture, not just educational techniques [20]. Lucey et al. (2018) call for a shift from performance-based to coaching-oriented mindsets [8], while Hawkins (2015) in medical education and Tovin (2022) in physical therapy education highlight the need for a paradigm shift toward accountability, flexibility, and integrated systems across the continuum [21,22]. Rhoney (2024) further underscores misalignment in pharmacy education between education, practice, and licensure, reflecting a lack of shared goals and language across systems [23]. This body of work suggests that CBHPE implementation has been constrained by entrenched paradigms that codify time-based progression, fragmented systems, and narrow conceptions of competence within training phases that require transformation.

These understandings led our author team to first explore the literature on systems interventions to understand the underlying drivers of systems performance that guide transformation. Second, this topic was prioritized at an all-day international in-person forum, where educators from around the world and across health professions discussed it in small and large groups to incorporate input from all ICBHPE participants. Third, we reviewed and discussed the literature to identify opportunities and barriers to a CBHPE continuum and to better understand HPE as a complex adaptive system. We identified multiple persistent barriers inhibiting a CBHPE continuum (Table 1). We then systematically identified themes within this literature that elucidate root causes of these barriers. Fourth, we identified Meadow’s framework of leverage points as informative for understanding CBHPE challenges. Leverage points are places within a complex system in which small interventions can have larger effects. We reviewed and discussed leverage points and collaboratively mapped observed and literature-based challenges onto a continuum within an HPE system, using Meadow’s framework of leverage points for intervening in systems, described in detail below and shown in Table 1 [11]. Fifth, we applied Meadows’s leverage points framework to link the identified CBHPE challenges to their corresponding levels of system influence. Categorization disagreements were resolved through iterative discussion until consensus was reached, ensuring shared interpretation across authors. This analytic process examined how different types of interventions may address systemic barriers to implementing a CBHPE continuum. Finally, the resulting analysis informed the systems-level recommendations presented below for strengthening CBHPE across the entire continuum.

**Table 1:** Barriers to a continuum of competency-based health professions education and root causes that align with Meadows’ leverage points*.


BARRIER	EXPLANATION OF BARRIER	ROOT CAUSE/DRIVER	LEVERAGE POINT GROUPING	SPECIFIC LEVERAGE POINTS

**Siloing across HPE continuum levels**	Lack of alignment among undergraduate/ graduate/ continuing education, with abrupt, disruptive transitions; leadership & governance structures remain separate and distinct.	Historical development of independent systems, separate program leaders, jurisdictional (governing authorities, regulatory bodies) control differences	Design Intent	Rules (governance structures), Goals (competing priorities across stages)

**Siloing within HPE continuum levels**	Schools, postgraduate education programs, and continuing professional development lack alignment even within the same stage/country, with inconsistent standards and approaches.	Decentralized control, institutional autonomy, lack of coordinating mechanisms	Design Intent	Rules (accreditation standards), Goals (institutional/program/department vs. system priorities)

**Time-based structures**	Fixed training durations, inflexible transition points (graduation, match), clinical service needs requiring uninterrupted coverage	Legacy systems prioritizing predictability, service delivery requirements, administrative efficiency.	Parameters Design	Rules (regulatory requirements), Parameters (duration requirements)

**Siloed assessment systems**	Non-integrated data platforms, technology disparities, privacy/fairness concerns about cross-stage information sharing, competition for limited specialist positions forces normative judgments	Technical infrastructure limitations, privacy regulations, institutional competition	Feedback Design	Feedback flows (assessment data), Rules (privacy regulations)

**Resource constraints and scarcity**	Limited training positions creating competition, insufficient faculty time for individualized approaches, inadequate funding for integrated systems and personalized progression	Finite resources (positions, faculty, funding), growing demand with program expansions, economic pressures, competing priorities for resource allocation	Parameters Design	Parameters (resource allocation), Rules (funding distribution), Goals (efficiency vs. individualization)

**Faculty development fragmentation**	Few educators are prepared to teach/assess longitudinally across the continuum; training focuses narrowly on a single stage; continuing professional development is often separate from faculty educator development.	Specialized expertise model, limited career incentives for cross-continuum work	Design Intent	Goals (faculty development priorities), Rules (promotion criteria)

**Limited individualized learning and progression capacity**	Faculty workload, clinical demands, scheduling constraints preventing accommodation of variable-rate learners	Resource constraints, efficiency pressures, traditional batch-processing model	Parameters Design	Parameters (resource allocation), Rules (scheduling systems)

**Misaligned accountabilities and outcomes**	Short-term performance priorities (graduate on time, match success), lack of longitudinal competence tracking, unclear readiness definitions	External pressure for immediate outcomes, measurement challenges, stakeholder expectations	Intent Feedbacks	Goals (accountability metrics), Feedback flows (outcome measurement)

**High-stakes examination requirements**	National board/licensing exams and maintenance of certification requirements anchor programs to time-based, summative judgments rather than longitudinal competence assessment	Regulatory gatekeeping function, standardization needs, public protection mandate	Design Intent	Rules (licensing requirements), Goals (standardization vs. individualization)

**Time-based funding models**	Government/institutional funding structured by credit hours, tuition by semester/year rather than competence achievement	Financial system architecture, budgeting predictability, administrative simplicity	Parameters Design	Parameters (funding formulas), Rules (financial regulations)

**Learner mindset and understanding**	Performance vs. growth orientation, fear of high-stakes transitions, inconsistent CBHPE implementation creating confusion	Assessment culture, high-stakes environment, implementation variability	Intent Feedbacks	Intent (learning culture), Feedback flows (assessment approaches)

**Cultural resistance and identity**	Adherence to tradition, educator/program identities focused on single stages, fear of unintended consequences.	Historical precedent, professional identity formation, risk aversion, change management challenges	Intent	Intent (fundamental beliefs about education), Goals (preservation vs. innovation)


*Meadows D. Leverage Points: Places to Intervene in a System. The Donella Meadows Project. 1999. https://donellameadows.org/archives/leverage-points-places-to-intervene-in-a-system/ (accessed 1 December 2025).

## Systems Thinking and Leveraging “Leverage Points” to Yield Recommendations

Systems thinking is a holistic approach to understanding problems that focuses on relationships, interactions, and patterns over time, rather than on isolated components or linear relationships, to explain and influence behavior in complex systems [24]. Achieving a CBHPE continuum requires a systems perspective, as persistent fragmentation reflects not only discrete barriers but also the interactions among structures, policies, and cultural norms. The CBHPE continuum is a complex adaptive system, defined as a system composed of multiple interacting agents that learn and adapt, producing emergent, nonlinear, and often unpredictable behavior at the system level [25]. CBHPE outcomes emerge over time from dynamic relationships among stakeholders, institutional governance, assessment practices, and regulatory environments [25,26,27]. Because of these interdependencies, interventions introduced in one part of the system often have unintended or attenuated effects. Isolated or technical solutions rarely produce sustained change without attention to the broader system [28].

Despite substantial investment, CBHPE implementation has produced limited transformation across the continuum of education to practice. Across health professions, misaligned structures for individualized progression, logistical constraints across training phases, and reliance on time-based (rather than competency-based) systems persist [8,21]. For example, learners who demonstrate readiness to advance face structural barriers to acceleration, while those who need more time may be stigmatized in performance-oriented cultures. These patterns suggest that many implementation challenges are downstream effects of deeper misalignments between competency-based principles and the systems in which they are enacted.

Meadows’ leverage points framework shifts attention to these underlying structures and patterns. We selected Meadows’ framework because it differentiates high- vs low-impact interventions in complex systems. It identifies high-impact opportunities to change system behavior by targeting root causes rather than surface-level symptoms. In the following section, we describe the leverage points framework and apply it to examine barriers to a CBHPE continuum and to identify strategic opportunities for system-level change. Meadows defines leverage points as places within a complex system where targeted interventions can produce substantial, sustained effects. This framework, which has been applied in fields from organizational management to environmental policy, helps explain why some interventions yield meaningful change while others do not [29,30].

Meadows’ hierarchy (shown in Table 2) distinguishes shallow and deep leverage points. Shallow leverage points are easier to modify but produce no or incremental improvements (‘tweaks to the system’). In contrast, deep leverage points (mindsets, paradigms, and goals) are harder to change but yield transformative effects across the system [11]. Aviation safety provides a useful example: modifying a checklist or adding a procedural step (a shallow, low-impact parameter) may yield small gains, but the major improvements in aviation safety emerged only when organizations reshaped deeper-level, high-impact assumptions about teamwork, communication, and shared responsibility for safety [31]. In HPE, interventions such as altering the number of assessments (parameters) to improve assessor workload and efficiency have limited impact on system behavior. In contrast, redefining readiness for practice (mindset) away from ‘time in training’ to ‘demonstrated competence’ can reshape expectations and practice across the entire HPE continuum [8,32].

**Table 2:** Meadows’ 12 Leverage Points* Applied to Health Professions Education.


MEADOWS’ LEVERAGE POINTS	DEFINITION SPECIFIC TO HEALTH PROFESSIONS EDUCATION	EXAMPLES FOR HEALTH PROFESSIONS EDUCATION

**Parameters: numbers, metrics. Easiest to change but least effective leverage points**.		

12. Constants, parameters, numbers	- Quantifiable aspects of the system that affect its performance but rarely transform its structure or goals. - Focus on numbers like budgets, staffing, or resource allocation.	- Number of trainees and teachers. - Number of clinical rotations. - Time spent on a rotation. - Amount of protected teaching time. - Annual budget for education. - Standardized test scores. - Per trainee funding. - Number of continuing professional development credits.

11. Sizes of buffers (something that cushions the impact of something else)	- Resource capacity to absorb disruptions in HPE without diminishing quality or outcomes. Too-small buffers increase risk; too-large buffers create inefficiencies.	- Having enough teaching faculty - Clinical site availability - Flexible room booking systems for teaching and clinical care. - Sufficient patients for trainees in different seasons

10. Structure of material stocks and flows and nodes of intersection (physical layout of the system and interconnections within the system)	- Physical and organizational infrastructure for HPE.	- Educational system covering undergraduate, graduate/postgraduate (or in some health professions, a single education phase) and continuing professional development phases. - Design and capacity of buildings (e.g., hospitals, clinics, universities). - Competing interests like patient care, education, and research. - Continuing professional development occurring off-site in conferences rather than directly within the work environment.

**Feedback: interactions within the CBHPE system, including positive and negative feedback loops**.		

9. Lengths of delays relative to rate of system change	- Timing mismatches between curricular or other education actions and observable results. Long delays prevent timely correction; short delays improve adaptability.	- Long delays between trainee performance and feedback (e.g., exam results weeks later). - Real-time dashboards for EPAs reduce lags. - Curriculum updates and subsequent change in trainee performance - Timely workplace-based assessments support immediate feedback for trainees. - Residency programs shortening promotion timelines after competency achievement. - Faculty feedback review and action plans reviewed at time of promotion rather than continuously.

8. Strength of negative feedback loops	- Balancing loops that self-correct the system. Weak loops fail to stabilize; strong loops promote individual and system resilience.	- Formative assessments guide trainee correction before summative failures. - Structured mentorship programs provide self-corrective mechanisms. - Accreditation requirements lead to program improvements - Remediation programs stabilize trainee progression. - Simulation case debriefs allow guided reflection and feedback.

7. Gain around positive feedback loops	- Reinforcing loops amplify growth or improvements in learners and programs.	- Awards, leadership roles, or scholarships support trainee growth. - Teaching awards motivate faculty teachers. - “Fast track” programs for high-performing trainees/residents. - Scholarly productivity leading to program reputational strength or trainees securing desired matches and faculty positions

**Design: structure and social structure of the CBHPE system**.		

6. Structure of information flows	- Changing access to information (feedback, performance data) transforms system behavior. Transparency, timely feedback, and distributed decision-making improve a competency-based education system.	- Transparent dashboards for EPAs, milestone progression, and competency tracking. - Real-time feedback from patients and clinical encounters shared with trainees. - Learning analytics tools identify struggling trainees early. - Peer-to-peer formative assessments. - Sharing performance data across sites to reduce siloed feedback. - Faculty teaching evaluations used in advancement decisions.

5. Rules of the system (incentives, constraints, policies)	- Policies, accreditation standards, advancement criteria, and financial incentives define what is possible in the system. Changing rules reshapes system behavior.	- Residency selection based on competency progression rather than fixed timelines. - Programmatic assessment policies including EPA entrustment rules. - Policy revisions to support remediation as part of learning and progression. - Protected time policies for preceptors and coaches. - Admissions reforms to reduce structural bias and foster inclusion. - Licensing exam policies.

4. Power to add, change, or self-organize system structure	- Ability of the system to evolve by creating new substructures, relationships, or novel components. Enables adaptive change and resilience.	- Trainees co-developing assessment rubrics and curricular content. - Development of distributed leadership teams in educational governance. - Emergence of longitudinal coaching roles. - Interprofessional faculty dyads and grassroots curriculum innovations. - Introduction of assessment tools and data analytics that use artificial intelligence (AI)

**Intent: underlying values and goals of the CBHPE system. Deepest, most impactful leverage points**.		

3. Goals	- The primary goal of the system is to improve health and healthcare, which determines its behavior and outcomes.	- Preparing trainees for team-based, patient-centered care. - Preparing trainees for AI-enriched clinical practice. - Supporting lifelong learning and adaptive expertise. - Encouraging trainees to practice in areas of societal need

2. Mindsets out of which system arises	- Fundamental beliefs, values, and assumptions that define system goals, structure, and rules. Changing mindsets requires letting go of traditions and old ways of thinking.	- Fixed vs growth mindset for trainees and educators. - Value placed on individual progress versus time-based progression. - Prioritization of interprofessional team-based care - Teacher as facilitator of learning rather than expert imparting knowledge (constructivist paradigm).

1. Power to transcend paradigms	- Ability to step outside prevailing paradigms and adopt new ways of thinking and operating. Challenges deeply held beliefs and fosters transformative change.	- Outcomes-based (vs. time-based) education. - Questioning the role of standardized exams and uses of scores. - Transforming the role of technology in education and health care - Quality and safety movements reshaping HPE.


*Meadows D. Leverage Points: Places to Intervene in a System. The Donella Meadows Project. 1999. https://donellameadows.org/archives/leverage-points-places-to-intervene-in-a-system/ (accessed 1 December 2025).

The leverage points framework has been consolidated into four larger areas, ranked from least to most impactful: parameters, feedback and information flows, design, and intent [33]. Examples of each area specific to HPE are listed here and elaborated in Table 2.

- **Parameters** (least impact, easiest to change): number of assessments, number of items on an assessment checklist, test scores required for a grade or for graduation, required faculty online CBHPE training modules.
- **Feedback and information flows** (moderate impact): feedback between a learner and a supervisor, learner formative assessment data prompting more individualized practice, learner success on assessments fed forward to their next rotation or next program, time between assessment activities and feedback to learners, learner or cohort data used by program leadership for evaluation, learner performance displayed for learners or program in a dashboard.
- **Design** (higher impact): policies addressing curricular time and program requirements, accreditation standards, program autonomy to design curriculum and assessment system versus requirements determined at national level.
- **Intent** (highest impact, hardest to change): changing from ‘time in rotations’ to demonstration of competence for advancement, shifting from value placed on knowledge exam scores to lifelong learning and clinical reasoning skills, changing assessment from end-of rotation evaluations to demonstration of competence with EPA-based assessment and awarding of micro-credentials [34], interactive faculty training about growth mindset.

Low-impact modifications fail to transform the system because the mental models and governing principles remain fixed.

The theoretical framework of leverage points offers guidance on where and how to intervene in complex systems, showing that not all interventions are equal. The most powerful changes come from altering system structure, goals, and underlying paradigms, rather than tweaking parameters. This approach reframes problem-solving in complex systems from “what action should we take?” to “which system intervention is most likely to produce transformative change?” Leverage points highlight epistemic (what counts as valid knowledge) and cultural factors as core drivers of system behavior. Therefore, a focus on leverage points encourages sequencing interventions to address paradigms such as adaptive expertise and goals such as lifelong learning skills early and continually. The leverage points framework reveals why many past HPE reforms fall short: they focus on low-impact intervention points such as creating new portfolio formats, adding more assessments, or requiring all faculty to complete one-time CBHPE training. These interventions do not address the underlying assumptions, rules, and mindsets that drive behavior regarding learning and assessment [35,36]. The CBHPE continuum requires targeting high-impact leverage points by fundamentally changing collective mental models about what constitutes readiness for practice, designing learning experiences to drive desired outcomes, and aligning system goals across the entire continuum of training and practice [37]. Applying Meadows’ framework [11] to CBHPE is a systems approach that moves beyond isolated tactical solutions to identify the most powerful points of intervention. These insights inform recommendations that address root causes, providing a comprehensive framework for systemic transformation toward a continuum of CBHPE.

## Vision for a Continuum of Competency-Based Health Professions Education

Informed by this understanding of the barriers and opportunities to achieving a CBHPE continuum, we propose a vision for it. This continuum can be strengthened by following recommendations shaped by an understanding of leverage points in complex systems. The vision for a CBHPE continuum is a seamless pathway that nurtures learners from students to unsupervised practice, ensuring they develop the skills and competencies needed to excel in their roles [10]. Table 1 highlights the specific structural, cultural, and regulatory misalignments that must be addressed to realize this vision. It also illustrates how deeper system-level interventions can transform fragmented elements into an integrated continuum. This continuum emphasizes a structured approach to assessments, integrating formative and summative feedback at every stage to foster continuous improvement and adaptability [2]. Learners experience transitions across rotations and levels of training as seamless continuations of their learning, allowing them to apply their knowledge in diverse contexts and refine their skills. Data-driven insights, personalized learning experiences, and frequent feedback contribute to a culture of lifelong learning, where health professionals are informed about their growing ability to demonstrate competence in real-world clinical settings.

While this vision may seem aspirational, we believe that health professions educators can make meaningful progress by applying competency-based principles and systems thinking, while collaborating across the education continuum. Ultimately, this vision aspires to enhance the quality and safety of patient care by producing well-rounded, reflective practitioners fully prepared to meet the evolving demands of the healthcare landscape. Although informed by literature review and discussion among an international collaborative, our analysis and examples are predominantly grounded in North American experiences, which may limit generalizability to global contexts.

## Recommendations

To operationalize this vision of the CBHPE continuum, we identify five system-level recommendations. Guided by Meadows’ leverage points framework [11], the recommendations progress from the most transformative areas of system intent and design to interventions that enhance information flows and feedback mechanisms.

### Recommendation 1: Target system architecture and structure (intent and design of the CBHPE system)

This foundational recommendation emphasizes governance alignment across the full CBHPE continuum for coordination and integration. Fundamentally restructuring how educational systems are organized and what they aim to achieve keeps the focus on clinical performance outcomes, from training to continuing professional development [38,39]. Addressing rules that govern assessment and advancement can enable seamless learner progression across the continuum. This requires designing a data architecture that enables learner progress data to flow across training levels and institutions, while addressing cultural resistance to information sharing and non-time-based progression.

Examples include creating opportunities for time-variable progression within programs and into practice, based on competence. Explicit entrustment protocols governing when learners can practice with decreasing supervision can enable the awarding of micro-credentials based on the defined skills needed for practice that flow from one stage of training to the next [34]. Restructuring assessment systems to replace episodic high-stakes exams with continuous programmatic assessment aggregates multiple low-stakes observations over time for well-rounded views of competence. Shifting from individual faculty grading to competency committees that make collective advancement decisions based on longitudinal data, and that follow learners from health professions school through residency, supports cohesive learning over time. Faculty development to enact graduated responsibility models in the learning environment where supervisory intensity adjusts dynamically based on demonstrated competence rather than training year enables individual progression. Redesigning governance and decision-making to create joint governance bodies across the continuum affords shared authority over curriculum design and learner progression. An aspirational goal would be to align governance across the health professions to promote team-based, interprofessional care.

### Recommendation 2: Prioritize interventions that shift mental models and paradigms (intent of the CBHPE system)

This foundational recommendation focuses on shifting mental models from ‘time served equals readiness’ to ‘demonstrated competence equals readiness.’ Achieving this shift requires the engagement of leaders and implementers in understanding CBHPE language and philosophy. The core components framework for CBHPE defines the essential elements of CBHPE that can be evaluated to determine the success of CBHPE as a transformative innovation, aligned with Meadows’ mindsets and paradigm shifts [4,40,41]. Shifting fundamental beliefs about how learning and competence should be demonstrated represents the most profound level of system change.

Examples include shifting from ‘time-in-training’ to ‘competence demonstrated’, and normalizing time-variable progression. To promote the belief that assessment exists to support growth, not primarily to sort or rank learners, programs can shift language from passing/failing to ‘ready/not yet ready’ [42]. Explicitly teaching that mistakes and setbacks are necessary for developing expertise, and sharing stories of practicing health professionals’ struggles that led to growth, encourage ongoing learning. A greater focus on professional identity formation shifts the emphasis from viewing competence as an individual achievement to collective, team-based care in which all members engage in lifelong learning and growth [43]. Fairness entails enabling individualized pathways to equitable opportunities for learning and the demonstration of competence. Inviting program-level experimentation and innovation around approaches that support a continuum can transform culture.

Supporting educators to reconceptualize their roles and identities as authorities who impart knowledge to coaches supporting learner development encourages them to design learning experiences that enable learners to develop and demonstrate competence. Supervisors then adopt the habit of mind of asking ‘what evidence do we have about this learner’s readiness for this specific activity?’

### Recommendation 3: Implement strategic high-impact, cross-continuum approaches (design, feedback and information flows)

This recommendation primarily addresses the leverage point of information flows, as it improves how information about learner progress and competencies moves through the system, enabling better insights, decision-making, and coordination of learning and promotion. It also addresses system design through rules and fundamental system organization (around competencies rather than around time-in-training experiences).

Examples include shared competency-outcomes language, such as the Foundational Competencies that align undergraduate and graduate medical education in the United States [44], and the alignment of EPAs across all levels, such as the EPAC (Education in Pediatrics Across the Continuum) initiative [45]. Implementing dashboards and portfolios with longitudinal learner analytics systems across the educational continuum can identify learners at risk of competency gaps, enabling greater learning supports [46,47]. Pairing shared competency language with technology systems that facilitate communication of competency achievement at one program or institution to populate learner records or dashboards at the next automatically facilitates continuous learning. Connecting learner competency data with patient outcomes across the educational continuum provides evidence of validity that competency assessments predict clinical performance. It also enables longitudinal tracking of competence across transitions from training to practice. This integration supports alignment of assessment standards across stages, informs developmental handovers, and creates feedback loops between education and clinical care that strengthen both learner progression and system-level quality improvement.

### Recommendation 4: Shift rules and incentives (design of the CBHPE system)

Educators collaborating with accrediting bodies to enable competency-based, flexible progression pathways should shift the rules and incentives that govern how the educational system operates. This recommendation targets Meadows’ leverage point of rules that govern the system, as changes to accreditation standards and institutional policies create new possibilities for how HPE functions. Though accreditation can drive focus on achieving outcomes, current accreditation practices and requirements vary globally, and some focus heavily on processes over outcomes [48]. Aligning expectations and reward structures with desired outcomes enables competency-based progression. Learners can advance based on demonstrated abilities, and institutions are incentivized to support time-variable progression.

Example innovations include promotion-in-place with sheltered supervision during residency to practice transitions [49] and accelerated health professional school programs that allow learners who demonstrate competence to progress more rapidly [50,51]. Realigning financial incentives to reward competency achievement and enable faculty to spend time with learners needing more practice to achieve competence addresses Meadows’ leverage point of changing the incentives that drive system behavior. Health professions school tuition models could charge the same amount regardless of how quickly students demonstrate competency, or graduate medical education could be adjusted to disincentivize hospitals from keeping trainees longer for service purposes. Accelerated progression financial incentives would reward institutions that successfully graduate health professionals based on competence [52]. Attention to the constraints in less-resourced programs and countries remains an important and unresolved challenge [53]. Shifting both rules and incentives creates the structural conditions necessary for a CBHPE continuum, where learners can seamlessly transition between educational phases based on demonstrated competence rather than arbitrary time boundaries.

Tying institutional or program accreditation to demonstrated competency outcomes rather than to process measures (such as the number of learning activities, curriculum hours, or faculty ratios) achieves outcomes-focused accreditation. Allowing time-variable progression in residencies officially recognized by specialty boards and creating maintenance-of-certification processes that use workplace-based assessment rather than separate recertification exams align competency-based outcomes and assessment approaches across the continuum. Health professions schools could provide promotion and tenure incentives by rewarding faculty for educational innovation in competency-based assessment and coaching, recognizing that CBHPE training and implementation requires significant time investment.

### Recommendation 5: Use data to inform feedback and improvement (feedback loops and information flows within the CBHPE system)

Continuously monitoring and refining the system based on evidence and outcomes creates iterative cycles of improvement, ensuring the CBHPE system evolves and adapts over time [54]. This recommendation establishes mechanisms for the system to learn about its own performance and make necessary adjustments to improve effectiveness.

Examples include building systems that match learners to clinical experiences based on data on their competency development needs, rather than on rigid rotation schedules. Combining individualized learning with systems that share learning information across sites and programs enables more customized learning. Implementing universal learner identifiers that persist across training institutions and into practice settings would support tracking of competency development and its linkage to patient outcomes. Data warehouses that aggregate competency achievement data for cohorts generate feedback to programs to improve their curricula.

Layered analysis is an approach to CBHPE program evaluation that examines educational innovations in three layers – techniques at the surface, principles in the middle, and philosophy at the core – to promote meaningful understanding of impact through specifying what was tried, under what circumstances, and what happened as a result [55]. Effective and ongoing evaluation provides valuable evidence for local implementers and quality assurance for institutional leaders, specialty organizations, and accreditors [56]. To support a continuum, program evaluation data must be shared forward and backward across phases of training. Evaluation of CBHPE programs should include learners’ and patients’ voices and provide evaluation data beyond individual course and clinical rotation evaluations [57].

## Conclusion

Achieving a CBHPE continuum requires coordinated system redesign across multiple interconnected HPE components. Realizing this vision demands moving beyond incremental, low-impact adjustments to embrace paradigm shifts in how we conceptualize learning, assessment, workforce development, and the institutional structures that support them. Innovative leadership from educators, administrators, accrediting bodies, professional organizations, policymakers, and healthcare systems is essential to dismantle siloed approaches and to implement a CBHPE continuum in practice.

We call upon stakeholders at all levels to commit to this transformative work. To realize a CBHPE continuum requires that educational institutions redesign foundational structures and cultures to support competency-based progression; healthcare organizations align workforce development practices with CBHPE principles; and funding agencies prioritize systems-level research and sustained implementation support. By intentionally addressing system elements including intent, design, and information flows within the leverage-points framework, we can ensure that interventions drive meaningful change throughout the education continuum. With coordinated, system-wide commitment, the field can realize the promise of a continuous, adaptive, and competency-based educational system that better serves learners, patients, and society’s evolving needs.

## Disclaimer

The opinions expressed in this article are those of the authors and do not necessarily reflect American Medical Association policy.
